# A simple vaporous probe with atomic-scale sensitivity to structural ordering and orientation of molecular assembly†
†Electronic supplementary information (ESI) available: Additional details for the experiments and data analysis. See DOI: 10.1039/c9sc01656b


**DOI:** 10.1039/c9sc01656b

**Published:** 2019-06-19

**Authors:** Han-Wen Cheng, Zhi-Peng Wu, Shan Yan, Jing Li, Shiyao Shan, Lichang Wang, Marc D. Porter, Chuan-Jian Zhong

**Affiliations:** a School of Chemical and Environmental Engineering , Shanghai Institute of Technology , Shanghai 201418 , China . Email: hwcheng@sit.edu.cn; b Department of Chemistry , State University of New York at Binghamton , Binghamton , New York 13902 , USA . Email: cjzhong@binghamton.edu; c Department of Chemistry and Biochemistry , Southern Illinois University , Carbondale , Illinois 62901 , USA; d Department of Chemistry and Chemical Engineering , University of Utah , Salt Lake City , Utah 84112 , USA . Email: marc.porter@utah.edu

## Abstract

Understanding the structural ordering and orientation of interfacial molecular assemblies requires an insight into the penetration depth of the probe molecules which determines the interfacial reactivity. In contrast to the conventional liquid probe-based contact angle measurement in which penetration depth is complicated by the liquid cohesive interaction, we report here a new approach that features a simple combination of vaporous hexane, which involves only van der Waals interaction, and quartz crystal microbalance operated at the third harmonic resonance, which is sensitive to sub-monolayer (0.2%) adsorption. Using this combination, we demonstrated the ability of probing the structural ordering and orientation of the self-assembled monolayers with a sensitivity from penetrating the top portion of the monolayers to interacting with the very top atomic structure at the interface. The determination of the dependence of the adsorption energy of vaporous hexane on the penetration depth in the molecular assembly allowed us to further reveal the atomic-scale origin of the odd–even oscillation, which is also substantiated by density functional theory calculations. The findings have broader implications for designing interfacial reactivities of molecular assemblies with atomic-scale depth precision.

## Abstract 2

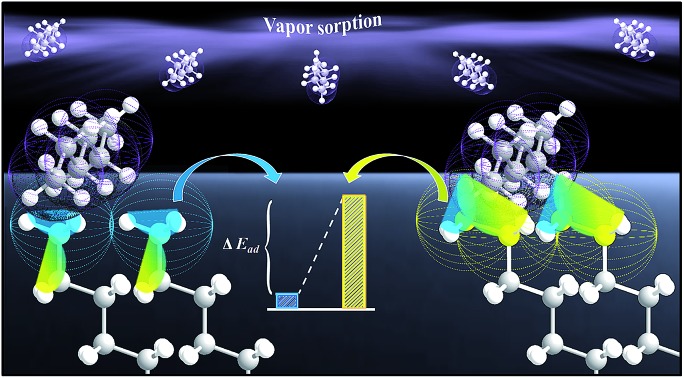
We report a simple combination of vaporous probe and quartz crystal microbalance with atomic-scale sensitivity to interfacial ordering and orientation.

## Introduction

Understanding the structural ordering and orientation of molecules on a surface, including atomically-flat single crystals and nanoscale-curved surfaces, is key to determining their interfacial functional properties.1a–d This understanding depends on the ability to probe the interface under *in situ*/*operando* conditions, which is however complicated often by the lateral intermolecular interactions of probing molecules, *e.g.*, contact angle and liquid-probe based measurements. The interaction of the probe and the surface-immobilized molecules determines the surface energetics for the structural ordering and orientation especially for molecular assemblies on surfaces such as self-assembled monolayers and other supramolecular structures. In addition to serving as a model system, the self-assembled monolayers of *n*-alkanethiolates on gold (CH_3_(CH_2_)_*n*_S/Au) continue to hold a strong interest of researchers in a wide range of phenomena,1e,f with applications in organic electronics,^2^ molecular-scale electronics,^3^ functional molecular junctions,4*a* and biofuel synthesis.4*b* These applications depend heavily on the structural ordering and orientation.

In particular, the preferential orientation of the all-*trans* alkyl chain structure depending on whether *n* is even or odd numbered has sparked a renewed interest in exploring molecular devices^5^ and technological impacts,^6^,^7^ including wettability,^6^ electrochemical charge transfer rates,^8^ electronic and vibrational surface spectroscopies^9^,1a and scanning probe microscopies.^9^ With vibrational sum-frequency generation spectroscopy, the sharp contrast in the odd–even oscillation between smooth and rough surfaces was believed to largely account for the opposing observations in the earlier works on wettability.^9^ By tunnelling current measurements through molecular junctions, the oscillation was linked to differences in the electrical resistance^7^ and the dielectric constant.^10^ The control of packing density and molecular orientation was also reported for other systems including selenium-based self-assembled monolayers and GaAs substrates.^11^ In addition to earlier friction force microscopy study of patterned domains of this system (*n* = 12–16)^12^ revealing a higher friction for the even-numbered monolayers, recent studies include STM probing of bi-component blends of isobutenyl compounds linked to long alkyl chains,^13^ and determination of charge transport across monolayers formed as molecular junctions on graphene.^14^ There is also in-depth study of substrate roughness dependent wettability measurements,^15^ where the impact of surface roughness on the odd–even structure was assessed by comparisons of wettability using as-deposited (Au^AD^) and smoother template-stripped (Au^TS^) substrates.^10^ The odd–even effect in hydrophobicity (water) was observed only on the smooth Au^TS^. The study of charge transport across self-assembled monolayers of *n*-alkanethiolates using junctions with the structure M^TS^/SAM//Ga_2_O_3_/EGaIn (M = Au or Ag, and EGaIn is eutectic GaIn) on Au^TS^ and Ag^TS^ showed statistically significant effect on Au^TS^, but not on Ag^TS^.^16^ A recent study of ferrocene-terminated alkyl thiol SAMs^17^ suggested that the dielectric constant or the polarity of the monolayer also plays a role in this structural effect. In molecular dynamics (MD) simulations,^18^ the calculated difference in interaction energy between odd- and even-*n* monolayer surfaces was 0.051 kcal mol^–1^ for hexadecane (HD), and almost zero for water, offering a partial account for some of the wetting studies of *n*-alkanethiolate monolayers.^9^ Traditionally, liquid probes such as water and HD were used to study the wetting properties, *e.g.*, contact angle measurements,^6^ but the strong probe–probe interaction in the liquid may influence the interaction of probe with the substrate. The liquid probe-based measurement involves unavoidable probe–probe cohesive energy, which differs between polar (*e.g.*, water) and non-polar liquids (*e.g.*, HD), thus complicating the measurement result.

**Scheme 1:**
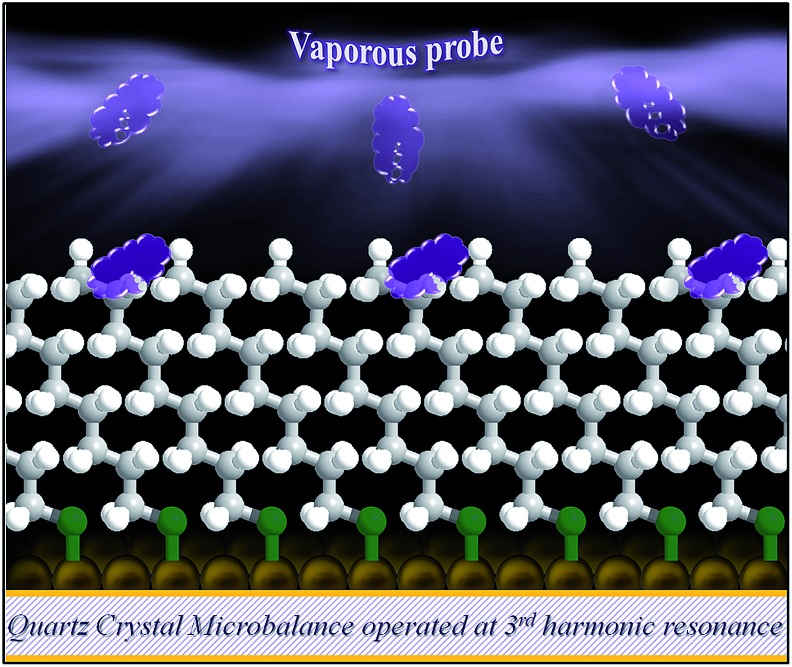
An illustration of the simple vaporous probe to terminal region of *n*-alkanethiolate self-assembled monolayer on gold (111) thin film coated quartz crystal microbalance operated at 3^rd^ harmonic frequency with a detection limit of 0.2% monolayer.

These complications show a clear need of a simple probe that can effectively detect the difference in degree of ordering or disordering, including rotational or *gauche* structures, in association with the penetration depth. We hypothesized that the surface adsorption of highly volatile hydrocarbons, *e.g.*, hexane (C_6_H_14_), would serve as a simple probe for determining the structural underpinnings through purely van der Waals interactions. This vaporous probe in combination with the highly mass sensitive quartz crystal microbalance (QCM) technique provides a simple platform for probing the structural ordering and orientation (see Scheme 1).

The weak van der Waals interaction between vaporous hexane molecule and the monolayer minimizes or eliminates the contributions from non-mass effects (*e.g.*, interactions by an adsorbate that changes the viscoelastic properties).^19^ We show that the QCM operating at the 3^rd^ harmonic frequency as opposed to the conventional fundamental frequency19*b* enables the detection of sub-monolayer adsorption of vaporous probe under only van der Waals interactions. This is critical for unravelling the energetics at the interface, which is in contrast to the traditional approach of liquid probes that could be complicated by strong intermolecular interactions (*e.g.*, contact angle measurement^6^). We present a simple technique to probe the interfacial structures without the complication of lateral interactions as in liquid probe used in contact angle measurement. We further show that our unique and simple approach allows detecting penetration depth at an atomic-level in terms of the preferential orientation of the alkyl chain structure with odd and even number of methylene units (*n*), which is to the best of our knowledge the first demonstration in unravelling the origin of the unique structural ordering and orientation of the molecular assembly.

## Results and discussion

**Fig. 1:**
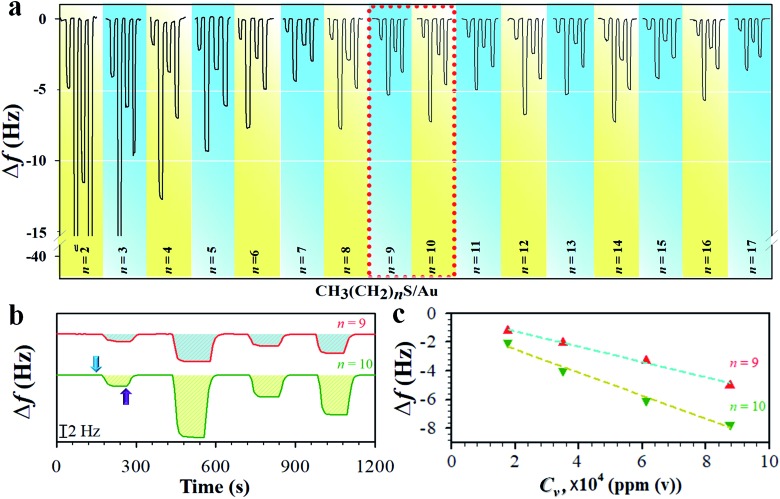
(a) Response profiles of frequency change (Δ*f*) *vs.* vapor concentration (*C*_v_, from left to right were 1.75, 8.74, 3.49, and 6.11 × 10^4^ ppm(v)) for hexane sorption on *n*-alkanethiolate monolayers (CH_3_(CH_2_)_*n*_S/Au) at 3^rd^ harmonic frequency (3*f*_0_). (b and c) The response profiles from the indicated area in a (b) and the corresponding plots of Δ*f vs. C*_v_ (c). The arrow indicates the onset of hexane flow (blue), and the start of N_2_ purges (purple).

We first determined the mass sensitivity (*S*_m_) of our QCM at both fundamental (*f*_0_) and 3^rd^ harmonic resonance frequencies (3*f*_0_) by the frequency change in response to the mass change in the formation of the monolayers (see Table S1†), substantiating the theoretical foundation. The change in frequency (Δ*f*) was then measured in response to the sorption of vaporous hexane on monolayers of different chain lengths. Fig. 1a shows a typical set of QCM responses at 3*f*_0_ for the monolayers when exposed to hexane at different vapor concentrations (see also the result at *f*_0_ in Fig. S1†). While the amplitude of the responses for shorter chains (*n* < 7) undergoes a gradual decrease, the amplitude for the longer chain monolayers (*n* ≧ 7) exhibits an oscillatory characteristic in terms of the overall frequency change. In Fig. 1b and c, two examples of the response profile and mass response sensitivity (Δ*f*/*C*_v_) are shown for *n* = 9 and 10. The hexane sorption is reversible, as reflected by the return of the response to its baseline upon re-exposure to a pure nitrogen gas stream. Δ*f* at both *f*_0_ and 3*f*_0_ is proportional to *C*_v_. The magnitude of Δ*f* at 3*f*_0_ is much larger than those at *f*_0_ (see ESI†). The overall magnitude of the response was also observed to decrease with chain length, indicative of the increase of the structural ordering.

**Fig. 2:**
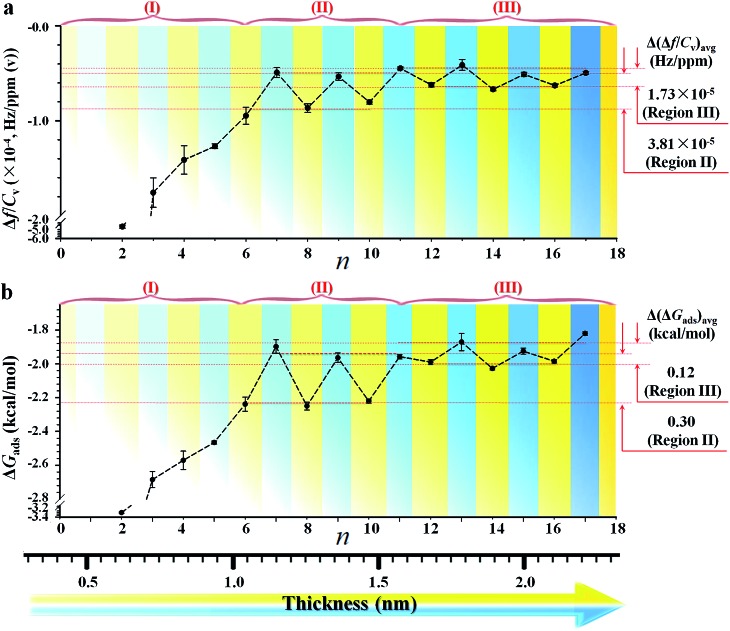
(a) Plot of the mass response sensitivity (Δ*f*/*C*_v_) *vs. n*, showing three regions (I, II, and III) with differences in terms of Δ(Δ*f*/*C*_v_)_avg_. (b) Plot of Δ*G*_ads_*vs. n*, showing differences in terms of Δ(Δ*G*_ads_)_avg_. Bottom scale illustrates the corresponding monolayer thickness in nm, ranging from 0.6 to 2.2 nm. This dependence was derived from at least three replicate sets of samples.

The mass sensitivity to hexane sorption, obtained from the slope of the plots of Δ*f vs. C*_v_ (Fig. 1c) displays a good linearity over the tested concentration range. The resulting ratio of *S*_m_ at 3*f*_0_*vs.* that at *f*_0_ (see Fig. S2 and Table S2†) is indeed close to 3, consistent with the classical predictions per equation in Table S1,† *i.e.*, the measurement at 3*f*_0_ is more sensitive than those at *f*_0_ by a factor of three. The signal-to-noise level at 3*f*_0_ translates to a sensitivity value as low as 0.2% monolayer of hexane (see Table S1 and footnotes†). The Δ*f*/*C*_v_, *i.e.*, slope (Fig. S2†), *vs. n*, as shown in Fig. 2a, exhibits distinctive features depending on the chain length. First, the magnitude exhibits a significant decrease with chain length up to *n* ∼ 6. Second, it displays an oscillatory pattern depending upon whether *n* is odd- or even-numbered. The even-*n* monolayers show a higher mass uptake than the odd-*n* ones. This dependence was observed for at least three replicate sets of samples. This oscillatory profile appears to show a larger magnitude for 6 < *n* < 11 than those with greater *n*.

Considering the adsorbed all-*trans* hexane with a fully extend molecular length or width (10.3 in length (*L*_Hx_) and 4.6 in width (*W*_Hx_), *i.e.*, 0.47 nm^2^ per hexane), an estimated coverage for a closest-packed adlayer of hexane would be 30.2 ng cm^–2^ (3.5 × 10^–10^ moles per cm^2^) (see Table S3†), which is quite close to the experimentally-determined monolayer coverage of hexane adsorption on graphite surface (0.46 nm^2^ per hexane).20*a* This value translates to a frequency change of 18.8 Hz for a full layer of hexane. In a recent determination of hexane vapor adsorption isotherm on the surfaces of mesoporous silica using a custom-built quartz spring microbalance,20*b* the isotherm is shown to be consistent with the cross-section area of hexane (0.52 nm^2^) and the monolayer adsorption (3.2 × 10^–10^ moles per cm^2^) (Table S3†), and sensitive to surface energy.

Since the hexane partial vapor pressure/saturation pressure ratio was lower than 0.6, a value needed for reaching hexane adsorption monolayer coverage,20*b* a sub-monolayer of hexane is more likely with the highly-ordered long chain monolayers. Thus, the actual coverage of hexane was estimated to be about 30% based on the average frequency change of ∼6 Hz for adsorption on *n* = 6–17 monolayers (Fig. 1a) *vs.* that for a full layer of hexane (18.8 Hz). The sub-monolayer adsorption suggests that there is no effective interaction among the adsorbed hexane molecules. The difference of response sensitivity between odd- and even-*n* monolayers (Δ(Δ*f*/*C*_v_)_avg_) depends on *n* in the three regions (I, II, and III) (see Fig. 2a). For Δ(Δ*f*/*C*_v_)_avg_ = 3.8 × 10^–5^ (II) and 1.7 × 10^–5^ (III) Hz ppm(v)^–1^, they correspond to a minimum of 0.7 (II) and 0.3 (III) Hz (Δ*f*_min_) or a maximum of 3.3 (II) and 1.5 (III) Hz (Δ*f*_max_). The Δ*f*_min_ translates to minimum Δ*m* of 1.1 × 10^–9^ (II) and 4.8 × 10^–10^ (III) g cm^–2^, whereas the Δ*f*_max_ projects to maximum Δ*m* of 5.4 × 10^–9^ (II) and 2.4 × 10^–9^ (III) g cm^–2^. Thus, the amount of hexane adsorbed on the even-*n* monolayers is greater than that on the odd-*n* monolayers by 4–18% in region (II) and 2–8% in region (III).

The above findings support the adsorption of vaporous hexane for probing the interfacial molecular orientation, which is further substantiated by adsorption kinetics and free energy analyses (see Fig. S3 and Table S4†). By fitting the kinetic data in terms of Langmuir adsorption isotherm, the equilibrium sorption constant, *K*, for hexane (see Table S5†) was found to range from 20 M^–1^ for *n* = 17 to 256 M^–1^ for *n* = 2. Interestingly, the value of *K* for the long chain monolayer is close to that (17 M^–1^) reported for hexane sorption on an activated carbon fabric adsorbent used for the separation and storage of VOCs.20*c* Using the determined values for *K*, the corresponding adsorption free energy (Δ*G*_ads_) for hexane was calculated (see Table S5†). The plot of Δ*G*_ads_*vs. n* (Fig. 2b) exhibits a trend similar to the Δ*f*/*C*_v_–*n* plot, depending on chain length. Δ*G*_ads_ is greater for adsorption on shorter chain monolayers (I). For longer chains, Δ*G*_ads_ is greater for even-*n* than odd-*n* monolayers (II and III).

The difference shows an adsorption free energy of the shorter chain being higher than that of the longer chain monolayers, reflecting likely disordering-enhanced sorption for the shorter-chain monolayers. Interestingly, these values are lower than the expectation based on the condensation energy of hydrocarbons (∼6 kcal mol^–1^),20b,c but very close to the cohesive energy reported for alkyl chains (*i.e.*, 1.4–1.8 kcal mol^–1^ (ref. 20*d*)). A key finding is that the average adsorption free energy difference between the odd- and even-*n* monolayers, Δ(Δ*G*_ads_)_avg_, is about 0.30 kcal mol^–1^ in region II and 0.12 kcal mol^–1^ in region III (Fig. 2b), a value quite close to the cohesive energy reported for the interaction between methylene units, *i.e.*, 0.2–0.8 kcal mol^–1^ of –CH_2_–.^1^,^9^ This cohesive energy reflected the adsorbate (hexane)–monolayer interaction without the complication of adsorbate–adsorbate interaction as in the case using liquid probe.^18^ The surfaces composed more of methylene groups have a greater negative free energy change than those of methyl groups. As shown in Fig. 2b, there is a much larger value of Δ*G*_ads_ for shorter chain monolayers. This adsorption free energy difference, *e.g.*, 1.5 kcal mol^–1^ between *n* = 2 and *n* = 15, reflects a higher accessibility of vapor probe to the underlying CH_2_ units as a result of the disordered packing, which agrees with IRRAS (Infrared Reflection Absorption Spectroscopy) data that indicate a liquid-like packing for short-chain monolayers and solid-like packing for long-chain monolayers.^9^

**Fig. 3:**
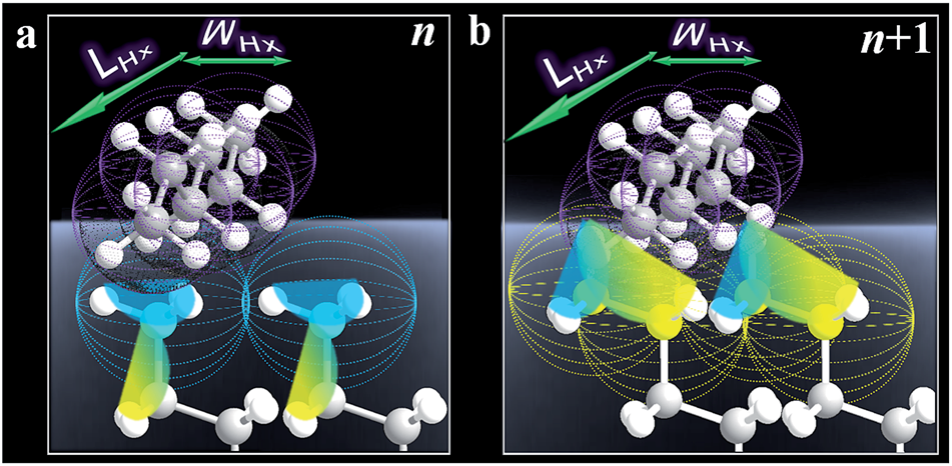
Illustrative adsorption of hexane with a molecular length (*L*_Hx_) and width (*W*_Hx_) on the very top layer of *n* (odd, a) and *n* + 1 (b) monolayers.

The fact that Δ(Δ*G*_ads_)_avg_ is close to the cohesive energy for methylene interactions suggests an enhanced accessibility of the vapor probe to the α-CH_2_ structure for the even-numbered monolayers. This gain in interaction energy is an important factor differentiating the methyl and methylene's interfacial free energies for adsorption. In sharp contrast to the findings from contact angle measurements, the vapor probe measurement provides a simple means to achieve high sensitivity to the top-most layer.^6^ For long-chain monolayers, the access of probe molecules deeper into the top-most layers is minimal. However, the presence of macroscopic disordering or defects diminishes odd–even phenomena, as in the case of less ordered or packed shorter chain monolayers, which is illustrated in Fig. 3 in terms of ordering and disordering for the different chain lengths (Fig. S4†).

Ideally, the all-*trans* model can be used to calculate the angle of methyl group for odd and even *n* by using the tilt angle of the alkyl chain with respect to the surface normal, *φ*, and the rotation angle of the alkyl chain, *ψ*.21*a* For *φ* = 30° and *ψ* = 50° with long-chain alkanethiol monolayers on Au (111), we have *θ* = 27° (odd *n*) and *θ* = 58° (even). The difference reflects a combination of changes in methyl angle (*θ*), angular distribution, and fraction of the *gauche* conformation at the terminal methylene unit (see Fig. S4†).

The strong van der Waals interaction between alkyl chains leads to a much lower fraction of the *gauche* conformation at the terminal for long-chain cases than that of an isolated alkyl chain. For *n* = 2–6, the changes in three structural parameters (*φ*, *ψ* and *θ*) are significant due to disordering regardless of *n*. The disordering overwrites the odd–even effect. For *n* = 6–11, the changes of three parameters are only significant for the even-*n* case,^21^ implying that this monolayer exposures more methylenes due to *gauche* structure. This is consistent with an extra van der Waals interaction energy of 0.69 kcal mol^–1^ in comparison with that for the *trans* conformation.21*a* For *n* > 11, the changes of the three parameters are not significant since only a few percent of *gauche* structures are present in the long-chain monolayers,^21^ correlating well with the oscillation of Δ*G*_ads_ in Fig. 2b.

The results demonstrated a clear difference in hexane penetration depth upon its adsorption on the monolayer (Fig. 2, 3, and S5†). For the shorter chain cases (*n* < 6), this penetration depth is large (up to ∼1 nm) due to disordering and *gauche* structures. For well-ordered long chain cases (*n* > 11), the penetration depth corresponds to the single terminal methyl group (∼0.2 nm) since the monolayer is free of *gauche* structure.

**Fig. 4:**
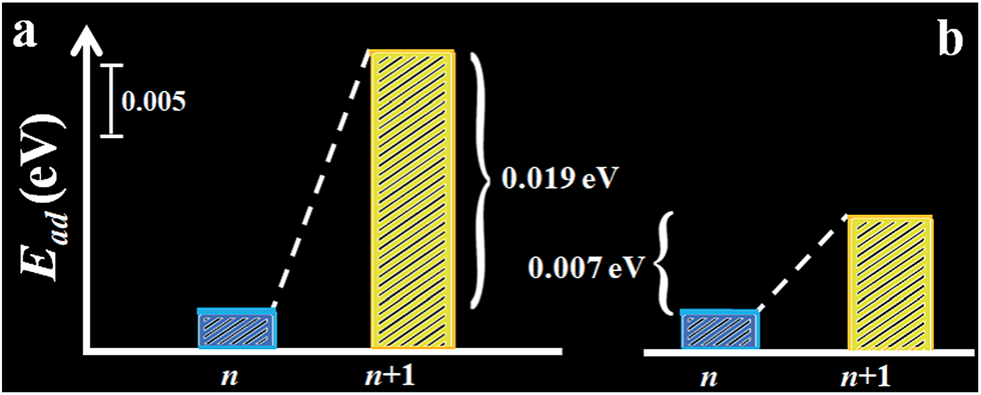
Plots of the calculated sub-monolayer (a) and monolayer (b) adsorption energy of hexane, *E*_ad_, on alkanethiolate monolayers (*n* = odd).

The assessment was further substantiated by density functional theory (DFT) calculation of the adsorption energy of hexane on the self-assembled monolayer (see Fig. 4 and Scheme S1†). Models of both monolayer and sub-monolayer adsorption of hexane were calculated, the latter of which ensures that there is no lateral inter-molecular interaction among hexane molecules. As shown in Tables S6 and S7,† the calculated hexane adsorption energies (*E*_ad_) oscillate between ∼0.05 eV for even-*n* monolayer and ∼0.04 eV for odd-*n*. The average difference of the adsorption energies of hexane between *n* and *n* + 1 monolayers is 0.01–0.02 eV, which translates to 0.23–0.46 kcal mol^–1^. This value is close to Δ(Δ*G*_ads_)_avg_ (0.30 (II) and 0.12 (III) kcal mol^–1^). It is remarkably consistent with the experimentally-observed trend, substantiating a higher adsorption energy of hexane on even-*n* than odd-*n* monolayers.

Note that the partial charge on S (–0.08 to –0.09*e*) and the Au–S bond length (2.545–2.555 Å) show little change before and after hexane adsorption. In agreement with the experimental data, the calculated adsorption energy features a lower value for the *n* (odd) monolayer and a higher one for the *n* + 1 monolayer.

Considering the dielectric constants or dipole moments for *n* and *n* + 1 monolayers^17^ (see Scheme S2†) and hexane is a non-polar molecule with low dielectric constant and only induced dipole-induced dipole interaction, we calculated the intermolecular potential between the monolayer and the hexane probe (see Fig. S6†). The subtle difference between *n* and *n* + 1 monolayers in interaction with hexane is consistent with the induced dipole-induced dipole interaction.

## Conclusions

In conclusion, we have demonstrated a simple and effective vaporous probe approach to unravelling the origin of the structural ordering and orientation of self-assembled monolayers. Our finding of the oscillatory characteristics of the adsorption energy between the monolayers of the different chain lengths closely reflects the van der Waals energy of hexane adsorption with a sensitivity from penetrating the top portion of the monolayers to interacting with very top atomic structure at the interface. This approach is not limited to hexane, and is in principle applicable to different combinations of monolayer surface and vapor probe properties by taking the separation of the specific mass effect into consideration. This unprecedented finding is further substantiated by theoretical calculations of the adsorption energies of hexane on the monolayers. In addition to an immediate implication of these findings to understanding the interfacial ordering and orientation on flat surfaces, a broader implication to the current research involving monolayer-capped gold nanoparticles and assemblies for various technological applications. Our finding of the vaporous interphase and interface sensitive probe may find applications in the exploration of molecular ordering and orientation using a wide range of other small molecules for chemical sensors^22^ and functional monolayer assemblies.^23^ The harnessing of the detailed molecular interactions on curved surfaces constitutes a frontier in designing gold nanoclusters with molecular precision,22a,^24^ and should attract a much broader interest of investigations.

## Experimental section

### Chemicals

(CH_3_(CH_2_)_*n*_SH, *n* = 2 to 17) and hexane (Hx, C_6_H_14_) as received (Sigma-Aldrich) were used. The QCM measurements used AT-cut quartz crystals (P. R. Hoffman Materials) with a 9 MHz (9.574 × 10^6^ Hz) fundamental resonance frequency. These crystals were 1.4 cm in diameter, 190.5 μm in thickness, and cerium-polished on both sides. Gold coatings were deposited in a “keyhole” shape by the resistive evaporation of gold (99.9% purity) onto both sides of the quartz disks, previously cleaned using piranha solution and rinsed with methanol, using an Edwards 306A cryopumped evaporator. The quartz disks were primed with 15 nm layer of chromium prior to gold deposition. The thickness of the gold film is 300 nm with the excitation electrode diameter of 0.48 cm. The pressure in the evaporator was less than 5 × 10^–6^ Torr before deposition. The roughness of the gold surfaces was similar to thin films deposited on glass substrates (Roughness Factor (RF): 1.3 ± 0.3).^25^

### Monolayer preparation

The gold-coated QCMs were cleaned in piranha solution (H_2_SO_4_ : H_2_O_2_ 3 : 1 v/v) and rinsed with purified water and ethanol multiple times before drying under a gentle stream of high purity argon (**Caution!** Piranha solution is highly reactive and should be handled with care in a fume hood). The cleaned QCM discs were carefully immersed into *n*-alkanethiol (1.0 mM in ethanol) solutions at room temperature (21.2 ± 0.2 °C) for 15 h. The resulting monolayers were rinsed thoroughly with ethanol and dried under argon.

### Hexane adsorption and QCM measurement

Hexane adsorption was performed at room temperature where the vapor pressure of hexane is 0.1747 bar. Nitrogen gas was used both as a reference gas and a diluent to manipulate the hexane vapor concentration. With a multi-gas controller (Model-147, MKS Instruments), flow rates of the hexane vapor stream were varied from 10 and 50 mL min^–1^ with N_2_ added at 100 mL min^–1^. Typical vapor concentrations for testing were 1.75, 3.49, 6.11 and 8.74 × 10^4^ ppm(v). The QCM device used gold coated on both sides, and was mounted in a water-jacked Teflon chamber with a conventional two-spring clip for electrical connection to a network analyzer (see Scheme S3†). Both sides of the QCM were exposed in the test vapor, which were accounted for when processing the data. The chamber was able to house to QCM devices, which allowed reference or cross comparisons in individual measurement.

A network analyzer (Model-HP8753C), with one-port scattering parameters (HP 85046A), was employed. Automatic data acquisition and analysis were performed using HP85165A resonator measurement software. The determination of the series resonance frequency (*f*), *i.e.*, the frequencies of maximum conductance, was conducted by measuring the electrical admittance over a range of frequencies centered about *f*_0_ or 3*f*_0_. The admittance data were then fit to an admittance circle with the Butterworth–VanDyke equivalent circuit model, from which the mass-loaded resonance frequency and a number of other circuitry parameters could be determined, including the series resistance *R*_1_ (energy dissipation), inertial inductance *L*_1_ (displaced mass), motional capacitance *C*_1_ (energy stored during oscillation), static capacitance *C*_0_, and quality factor *Q* (energy stored *vs.* energy loss). Measurements were carried out at both *f*_0_ (9 MHz) and 3*f*_0_ (27 MHz), most of the reported data were collected at 3*f*_0_, which has a higher mass sensitivity. The oscillator circuit parameters, *R*_1_, *L*_1_, *C*_1_, and *C*_0_, were fit in the admittance plane locus to obtain *Q* > 10^5^ at *f*_0_ and 3*f*_0_. The uncertainties in the determined values for *R*_1_ and other equivalent circuit parameters were ∼5%.^26^ Details for the experimental set up and data analysis are described in ESI (Table S1 and Scheme S3†).

### Computational modeling

DMol package in the Materials Studio Software was used for the periodic DFT (density functional theory) calculation. The calculation involved Perdew–Burke–Ernzerhof (PBE) function with a generalized gradient approximation (GGA) for the exchange–correlation interaction. Au(111) is used as the substrate surface for the adsorption of alkanethiolate molecules. The adsorption energy of hexane on SAM/Au(111) was calculated by *E*_ads_ = –(*E*_Hx-SAM/Au(111)_ – *E*_SAM/Au(111)_ – *E*_Hx_), where *E*_Hx-SAM/Au(111)_, *E*_SAM/Au(111)_ and *E*_Hx_ are total energy for the Hx-SAM/Au(111), the SAM/Au(111), and the isolated hexane molecule, respectively.

## Conflicts of interest

There are no conflicts of interests to declare.

## Supplementary Material

Supplementary information

## References

[cit1] Nerngchamnong N., Yuan L., Qi D. C., Li J., Thompson D., Nijhuis C. A. (2013). Nat. Nanotechnol..

[cit2] Casalini S., Bortolotti C. A., Leonardi F., Biscarini F. (2017). Chem. Soc. Rev..

[cit3] Xiang D., Wang X., Jia C., Lee T., Guo X. (2016). Chem. Rev..

[cit4] Seo S., Hwang E., Cho Y., Lee J., Lee H. (2017). Angew. Chem., Int. Ed..

[cit5] Thompson D., Nijhuis C. A. (2016). Acc. Chem. Res..

[cit6] Newcomb L. B., Tevis I. D., Atkinson M. B., Gathiaka S. M., Luna R. E., Thuo M. (2014). Langmuir.

[cit7] Jiang L., Sangeeth C. S., Nijhuis C. A. (2015). J. Am. Chem. Soc..

[cit8] Baghbanzadeh M., Bowers C. M., Rappoport D., Żaba T., Yuan L., Kang K., Liao K. C., Gonidec M., Rothemund P., Cyganik P., Aspuru-Guzik A., Whitesides G. M. (2017). J. Am. Chem. Soc..

[cit9] Walczak M. M., Chung C., Stole S. M., Widrig C. A., Porter M. D. (1991). J. Am. Chem. Soc..

[cit10] Tao F., Bernasek S. L. (2007). Chem. Rev..

[cit11] Shaporenko A., Müller J., Weidner T., Terfort A., Zharnikov M. (2007). J. Am. Chem. Soc..

[cit12] Wong S. S., Takano H., Porter M. D. (1998). Anal. Chem..

[cit13] Kikkawa Y., Tsuzuki S., Taguchi K., Kashiwada A., Hiratani K. (2017). Phys. Chem. Chem. Phys..

[cit14] Song P., Thompson D., Annadata H. V., Guerin S., Loh K. P., Nijhuis C. A. (2017). J. Phys. Chem. C.

[cit15] Wang Z., Chen J., Oyolareynoso S., Thuo M. M. (2016). Langmuir.

[cit16] Baghbanzadeh M., Simeone F. C., Bowers C. M., Liao K. C., Thuo M. M., Baghbanzadeh M., Miller M. S., Carmichael T. B., Whitesides G. M. (2014). J. Am. Chem. Soc..

[cit17] Feng Y., Dionne E. R., Toader V., Beaudoin G., Badia A. (2017). J. Phys. Chem. C.

[cit18] Srivastava P., Chapman W. G., Laibinis P. E. (2005). Langmuir.

[cit19] Martin S. J., Granstaff V. E., Frye G. C. (1991). Anal. Chem..

[cit20] Arnold T., Thomas R. K., Castro M. A., Clarke S. M., Messe L., Inaba A. (2002). Phys. Chem. Chem. Phys..

[cit21] Nishi N., Hobara D., Yamamoto M., Kakiuchi T. (2003). J. Chem. Phys..

[cit22] Cheng H. W., Wang J., Li Y. J., Li J., Yan S., Shan S., Wang L., Skeete Z., Zhong C. J. (2018). Small.

[cit23] Krzykawska A., Szwed M., Ossowski J., Cyganik P. (2018). J. Phys. Chem. C.

[cit24] Reimers J. R., Ford M. J., Marcuccio S. M., Ulstrup J., Hush N. S. (2017). Nat. Rev. Chem..

[cit25] Walczak M. M., Lamp B. D., Alves C. A., Porter M. D. (1995). J. Electroanal. Chem..

[cit26] Shinar R., Liu G. J., Porter M. D. (2000). Anal. Chem..

