# Supplementary material for: A simple vaporous probe with atomic-scale sensitivity to structural ordering and orientation of molecular assembly
Source: Chem Sci. 2019 Jun 19;10(29):7104–10. doi: 10.1039/c9sc01656b (PMC6677115; doi:10.1039/c9sc01656b)
Supplement: Supplementary file 1 [file SC-010-C9SC01656B-s001.pdf]

Electronic Supplementary Material (ESI) for Chemical Science.
This journal is © The Royal Society of Chemistry 2019

Electronic Supplemental Information

A Simple Vaporous Probe with Atomic-Scale Sensitivity to Structural Ordering and Orientation of Molecular Assembly

Han-Wen Cheng,*[a],[b] Zhi-Peng Wu,[b] Shan Yan,[b] Jing Li,[b] Shiyao Shan,[b] Lichang Wang,[c] Marc D. Porter,*[d] and Chuan-Jian Zhong*[b]

[a] School of Chemical and Environmental Engineering, Shanghai Institute of Technology, Shanghai 201418, China

[b] Department of Chemistry, State University of New York at Binghamton, Binghamton, New York 13902, USA

[c] Department of Chemistry and Biochemistry, Southern Illinois University, Carbondale, Illinois 62901, USA

[d] Department of Chemistry and Chemical Engineering, University of Utah, Salt Lake City, Utah 84112, USA

Supplementary Tables and Figures:

Table S1. Comparisons of the theoretical and experimental Δ values from the measured values of frequency changes (Δ*f*) from alkanethiolates with two different chain lengths (*n*=17 and 3) formed on gold coated QCMs. The difference in the frequency changes ($\Delta f_{n=17}$ - $\Delta f_{n=3}$) corresponds to the mass of $–(CH_2)_{14}–$.

$\Delta f_{n=17} - \Delta f_{n=3}$ (Hz)[a)]	Theoretical Δm (g/cm^2)[b)]	Experimental Δm (g/cm^2)[c)]
-32.2 ± 4.1 (N=1)	1.76×10^{-7}	$(1.55 \pm 0.20) \times 10^{-7}$
-93.7 ± 5.3 (N=3)	1.76×10^{-7}	$(1.50 \pm 0.09) \times 10^{-7}$

[a)] N: order of harmonic resonance frequency; [b)] 9.0×10^{-10} moles/cm² × 196 g/mol; [c)] based on Eqn. 1: for N=1, use $S_m = 2.08 \times 10^8$ Hz cm²/g, and for N=3, use $S_m = 6.23 \times 10^8$ Hz cm²/g.

The most critical parameter to validate the advantage carrying out QCM measurements at $3f_0$ is mass sensitivity. Under ideal conditions (*i.e.*, the acoustic impedances of the crystal and adsorbed materials are identical), the relationship between frequency change, Δf (Hz), and the mass change, Δm (g/cm²), due to surface adsorption is given by the Sauerbrey equation:[1]

$$\Delta f = -\frac{2Nf_0^{\,2}}{\sqrt{\rho_q \mu_q}}\Delta m = -S_m \Delta m \quad \text{(Hz)} \qquad \text{(Eqn. 1)}$$

where $\Delta f = f - f_0$, f_0 is the fundamental resonance frequency (9.574×10^6 Hz) of the QCM, N is the number of the harmonic used to drive the QCM (odd-number integers only), ρ_q is the density of AT-cut quartz (2.648 g/cm³), and μ_q is the shear modulus of AT-cut crystal (2.947×10^{11} g/(cm·s²)). The negative sign recognizes that a decrease in resonance frequency reflects an increase in mass loading. As previously detailed,[2] the proportionality connecting Δf and Δm, the mass sensitivity (S_m), can be defined by normalizing the differential sensitivity (d*f*/d*m*) to f_0, *i.e.*, d*f*/[f_0(d*m*)] to yield $S_m = 2Nf_0^{\,2}/\sqrt{\rho_q \mu_q}$ (Hz cm²/g). Theoretically, the value of S_m equals 2.08×10^8 Hz cm²/g (0.208 Hz cm²/ng) for the 9 MHz fundamental mode and 6.23×10^8 Hz cm²/g (0.623 Hz cm²/ng) for the 27 MHz third harmonic mode. Operation at $3f_0$ has a larger S_m than that at f_0, which can be achieved by thinning the quartz crystal but is experimentally difficult to use.[1] The values of S_m at f_0 and $3f_0$ can be experimentally determined by the responses to monolayer formation and the responses to vapor sorption. The fundamental frequency (f_0) is related to the crystal thickness (t_q) by $f_0\ t_q = v_q/2$, where the quartz crystal's linear velocity $v_q = 3.34 \times 10^5$ cm/s. For f_0 =9 MHz, t_q is 186 μm, whereas for f_0 =27 MHz, t_q is 61 μm. From operational perspective, the routine use of a stand-alone crystal disk with a 60-μm thickness may be possible, as demonstrated in an earlier study using QCM of 50-μm thickness with a fundamental frequency of 30 MHz for achieving a high detection sensitivity.[1] However, such QCM was also found to display an enhanced sensitivity to viscosity[1], which is not desired for pure mass sensitivity as needed for the present work. The result compares the theoretical and the experimental values of Δm determined from the Δf measured for alkanethiolate monolayers with two different chain lengths (*n*=17 and 3). These data show ~3% difference in the mass change found for measurements at f_0 with respect to that at $3f_0$. Indeed, the measured frequency change ($\Delta(\Delta f)$) is only ~13% lower than that expected theoretically, which we attribute to the more liquid like-nature (*i.e.*, lossy) of the short chain SAM that led to a more disordered monolayer in comparison with the highly-ordered nature of the long-chain SAM. These results indicate that our measurements are capable of detecting mass changes at the sub-monolayer level. Analysis of the noise of measured frequency responses yielded a standard deviation (SD) of 0.010 Hz for the operation at f_0 and 0.013 Hz for the operation at $3f_0$. Using $\Delta m_{min.} = \Delta f_{min.}/S_m$, the minimum signal ($\Delta f_{min.}$), 3 × SD, translated to LOD of 0.144 ng/cm² (3 × 0.010 Hz/0.208 Hz cm²/ng) for the operation at f_0 and 0.063 ng/cm² (3 × 0.013 Hz/0.623 Hz cm²/ng) for the operation at $3f_0$.

Considering that there is frequency change of 18.8 Hz for one monolayer of hexane adsortion on the OCM operating at $3f_0$ (see manuscript) and the 3×SD (i.e., 3x0.013 Hz), the LOD for detection of hexane adsortion is 0.2% monolayer of hexane, a value corresponding to a very small sub-monolayer level.

Note that this measurement is not limited to hexane vapor. Other small gas or vapor molecules with polar surfaces can be used. We selected vapor molecules based on the consideration of interactions such as van der Waals, polarity, and hydrogen bonding. We have in fact tested ethanol and water vapors. In these cases, the adsorption process is still reversible. The adsorption and desorption kinetics depend on the vapor type and concentration. Weaker interaction leads to smaller response. Stronger polar and hydrogen bonding interactions lead to longer response time. The only complication is the elasticity effect in addition to mass effect. In the case of only van der Waals interaction, the viscoelasticity is not operative. With stronger interactions, there are two methods to deal with the viscoelastic effect. One involves lower vapor concentrations so that the adsorption is limited to sub-monolayer level where the viscoelastic effect is insignificant. The other method involves admittance or impedance analysis of the oscillation circuit where

the mass and viscoelastic effects could be separated. In summary, this approach is in principle applicable to many other cases but complications must be considered in terms of methods in applying the technique for different combinations of monolayer surfaces and vapor probe properties.

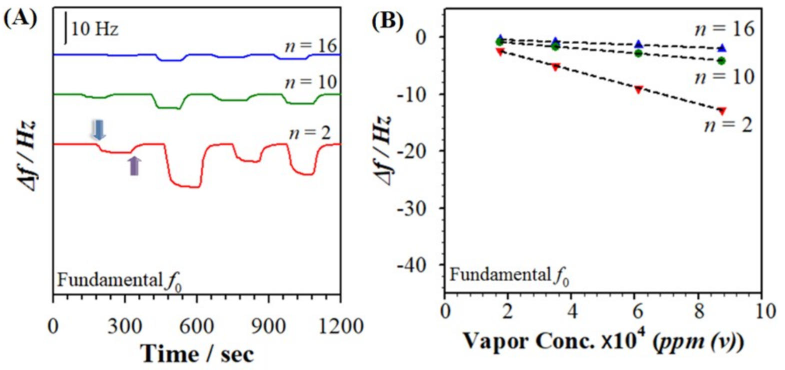

Fig. S1. Frequency change response profiles (A) and frequency change *vs*. vapor concentration plots (B) for hexane sorption on alkanethiolate monolayers of the indicated chain lengths ($CH_3(CH_2)_nS/Au$) at fundamental frequency f_0. The hexane vapor concentrations (left to right): 1.75, 8.74, 3.49, and 6.11×10^4 ppm(v). The arrow indicates the onset of hexane flow (blue), and the start of N_2 purges (purple). Dashed line (B): linear regressions.

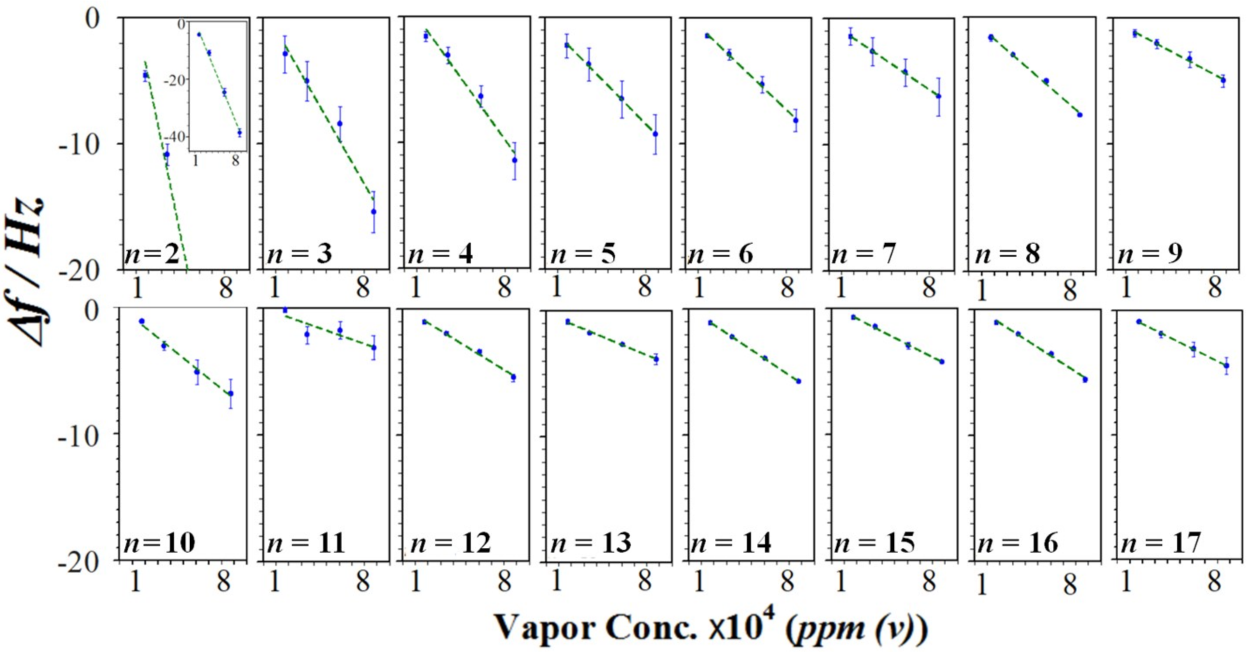

Fig. S2. Dependence of frequency change on the vapor concentrations of hexane for alkanethiolate monolayers of the indicated chain length (*n*) at 3rd harmonic frequency ($3f_0$). Lines: linear regression with the slope representing the mass sensitivity. Similar to the slope – *n* oscillatory behavior in Fig. 2a, the intercept – *n* plot showed a oscillatory behavior from the shorter chain up to *n* =11, after which it diminishes. The fact that there is a near-zero intercept for the long chain monolayers suggests that the adlayer's elasticity is negligible, supporting that the measured frequency changes for hexane vapor sorption are controlled by mass loading and not affected by the elasticity complications often encountered with sorption of polar molecules. Both the slopes and the intercepts of the linear regressions showed an oscillatory behavior.

Table S2. Ratio of mass sensitivity at $3f_0$ *vs*. f_0 (S_{3f_0}/S_{f_0}) as determined for alkanethiolate monolayers with different *n* in $CH_3(CH_2)_nS$-Au.

n in $CH_3(CH_2)_nS$-Au	S_{3f_0}/S_{f_0}	
	Theoretical	Experimental
2	3.00	3.24 ± 0.13
10	3.00	3.07 ± 0.11
16	3.00	2.93 ± 0.08

Table S3. Area per hexane molecule (nm^2/#) and monolayer coverage (moles/cm^2) based on literature reports and model-derived values.

Values reported in Refs.						Model of hexane in this work ****)	
Ref 5*)		Ref 6**)		Ref 7***)			
nm^2/#	mol/cm^2	nm^2/#	mol/cm^2	nm^2/#	mol/cm^2	nm^2/#	mol/cm^2
1.21	1.37×10^{-10}	1.07	1.54×10^{-10}	0.52	3.19×10^{-10}	(1.03 × 0.46) 0.47	3.50×10^{-10}

* Note: based on hexane adsorption on Activated Carbon Fiber in supercritical CO_2 with a density of 0.32 g/cm^3.[3]
** Note: based on hexane adsorption on Activated Carbon Cloth in water.[4].
*** Note: based on hexane adsorption on lyophobic mesoporous silica (SBA-15 ordered mesoporous silica functionalized with lyophobic perfluoroalkyl groups).[5]
**** Note: based on all trans structural model with 10.3 Å in length and 4.6 Å in width,[6] and (111) packing of the hexane molecules on the surface.

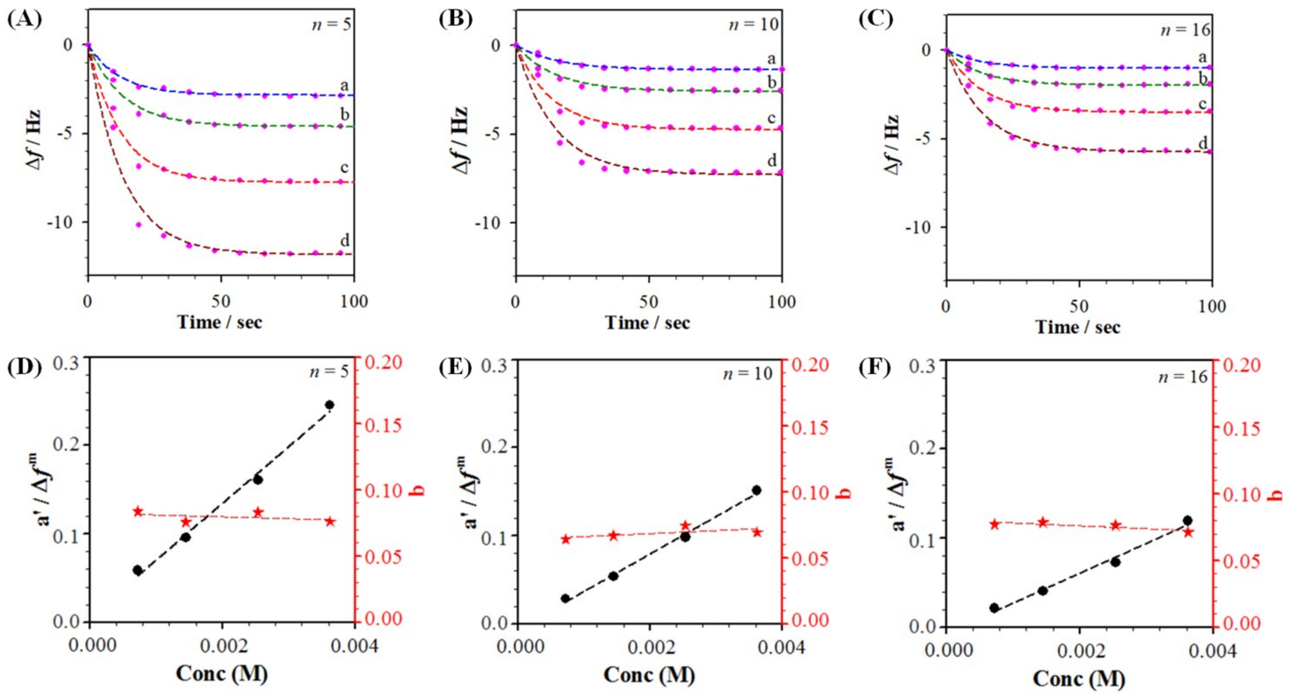

Fig. S3. Transient frequency change response profiles upon adsorption of hexane (A-C) at different vapor concentration (mol/L, $\times 10^{-3}$): 0.725 (a), 1.45 (b), 2.54 (c) and 3.62 (d) of different alkanethiolate ($CH_3(CH_2)_nS$/Au, n = 5 (A), 10 (B) and 16 (C)). Dashed lines: the theoretical fit based on Langmuir adsorption isotherm (Eqn. 2, generating fitting parameters a and b; all curves display excellent fits, as reflected by $R^2 > 0.980$, standard error of estimate <0.50 Hz, and residual's mean square (MS) < 0.2 Hz for all transient data). (D-F) Plots of values of a' and b *vs*. hexane concentration (mol/L) for n=5 (D), n=10 (E), and n=16 (F). For a' ~ conc. curves (black), dashed lines represent linear regression (see Table S4 for slopes and intercepts). For b ~ conc. curves (red), the slopes are close to zero and the intercept are close to 0.07. The rate of adsorption is assumed to be not controlled by diffusional mass transport to the monolayer surface, and the uptake and loss of hexane are assumed to obey a Langmuir adsorption isotherm, which are reasonable for processes of the vapor sorption involving only hydrophobic interactions.[42,43] The fractional surface coverage (θ, where $\theta = \Delta f_t/\Delta f^{m}$) at a given vapor concentration can then be expressed as: $\theta = a[1-\exp(-bt)]$, where $a = C_M/(C_M + K^{-1})$, $K = k_f/k_b$, and $b = k_f C_M + k_b$ (Eqn.2). Δf_t and Δf^{m} are the frequency changes at time t and at full hexane adlayer coverage, respectively, K is equilibrium constant for hexane sorption, and C_M is the molar concentration of hexane vapor. The above equation can also be written as: $\Delta f_t = a' \times [1-\exp(-bt)]$,where $a' = \Delta f^{m} \times a$ (Eqn.3).

Table S4. Linear regression (Fig. S3 D-F) slopes and intercepts.

n in $CH_3(CH_2)_n$S-Au	Slope (M^{-1})	Intercept (dimensionless)	Linear fitting parameter (R^2)
5	64.31	6.11×10^{-3}	0.9919
10	42.52	-5.65×10^{-3}	0.9956
16	33.59	-6.50×10^{-3}	0.9883

Table S5. The equilibrium constant (K) estimated from hexane adsorption.

n in $CH_3(CH_2)_nS$-Au	K (M^{-1})	ΔG_{ads} (kcal/mol)
2	256.26	-3.28
3	93.23	-2.68
4	76.87	-2.57
5	64.31	-2.46
6	43.85	-2.24
7	24.67	-1.90
8	44.67	-2.25
9	27.57	-1.96
10	42.52	-2.22
11	27.28	-1.96
12	28.78	-1.99
13	23.563	-1.87
14	30.67	-2.03
15	25.81	-1.92
16	28.57	-1.98
17	21.72	-1.82

Note: Using the determined values for K, the corresponding adsorption free energy (ΔG_{ads}) for hexane on the monolayer surface was calculated according to $\Delta G_{ads} = -RT\ln K$

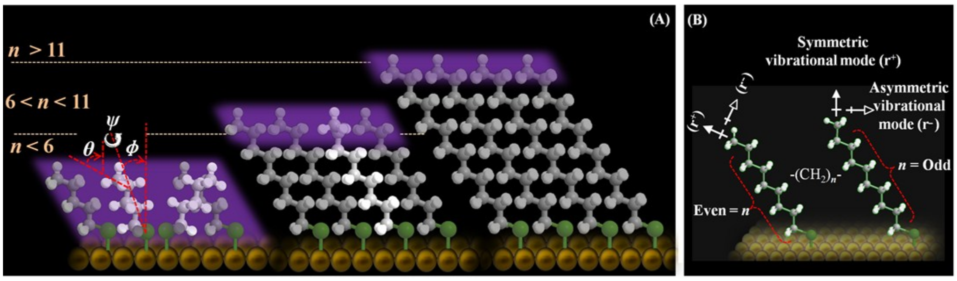

Fig. S4. (A) Schemes showing ordering and orientation for monolayers with three chain-length regions I (n= 2~6, thickness <1.0 nm), II (n= 6~11), and III (n >11, thickness >1.6 nm); and Illustration of the combination of changes caused by *gauche* conformation, including the change in the methyl angle (θ), the tilt angle of the alkyl chain with respect to the surface normal (ϕ), and the rotation angle of the alkyl chain (ψ), as indicated; The highlighted areas illustrate the average vapor penetration depth. (B) A schematic view of the all-trans model in terms of the average orientations of the methyl groups CH_3 vibrates symmetrically (r^+) or asymmetrically (r^-), displaying a distinctive difference for odd and even n case.

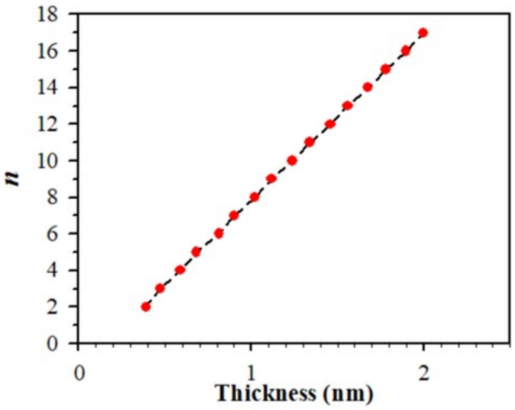

Fig. S5. Plot of chain length (n) *vs*. the nanoscale thickness of the monolayers.

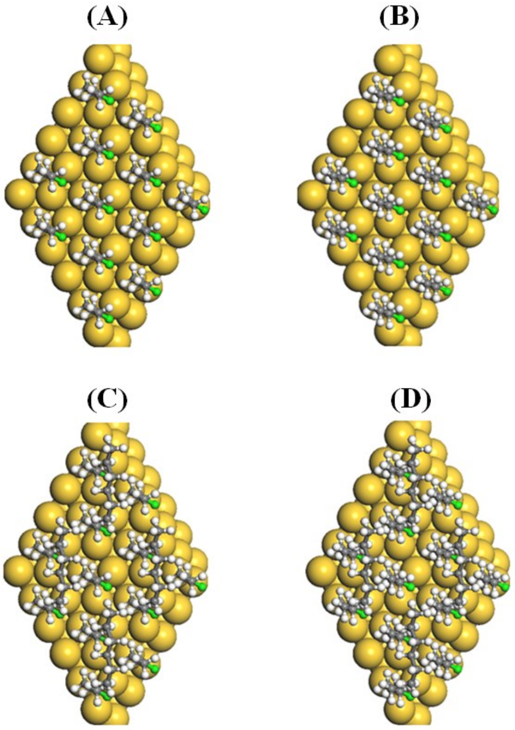

Scheme S1. Models for " even-*n*" $CH_3(CH_2)_2S$ /Au(111) (A), and "odd-*n*" $CH_3(CH_2)_3S$ /Au(111) (B); and for adsorption of hexane on $CH_3(CH_2)_2S$ /Au(111) (C), and on $CH_3(CH_2)_3S$ /Au(111) (D), in the DFT calculations. (slab model: 3×3 unit cell)

Table S6. DFT calculated results for the adsorption of hexane on odd- and even-numbered monolayers on Au (111).

Hexane adsorption	*n*	E_{ad} (eV)
Hexane on $CH_3(CH_2)_2S(-)$ /Au	even	0.049
Hexane on $CH_3(CH_2)_3S(-)$ /Au	odd	0.042
Hexane on $CH_3(CH_2)_4S(-)$ /Au	even	0.049
Hexane on $CH_3(CH_2)_5S(-)$ /Au	odd	0.043
Hexane on $CH_3(CH_2)_2S(-)$ /Au	even	0.028
Hexane on $CH_3(CH_2)_2S(-)$ /Au	odd	0.009

Table S7. DFT calculation results for self-assembled monolayers on Au (111).

Self-assembly monolayer on Au (111)	*n*	E_{ad} (eV)
$CH_3(CH_2)_2S(-)$ /Au(111)	even	1.51
$CH_3(CH_2)_3S(-)$ /Au(111)	odd	1.53
$CH_3(CH_2)_4S(-)$ /Au(111)	even	1.52
$CH_3(CH_2)_5S(-)$ /Au(111)	odd	1.53

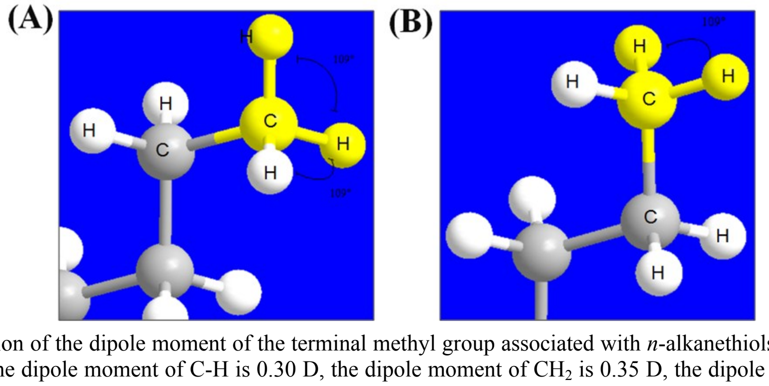

Scheme S2. Models for calculation of the dipole moment of the terminal methyl group associated with *n*-alkanethiols ($CH_3(CH_2)_nSH$, *n*=even (A), and *n*=odd (B)). As reported[7], the dipole moment of C-H is 0.30 D, the dipole moment of CH_2 is 0.35 D, the dipole moment of CH_3 is 0.33 D (B, *n*=odd), and the dipole moment of CH_2-CH_3 is 0.34 D (A, *n*=even).

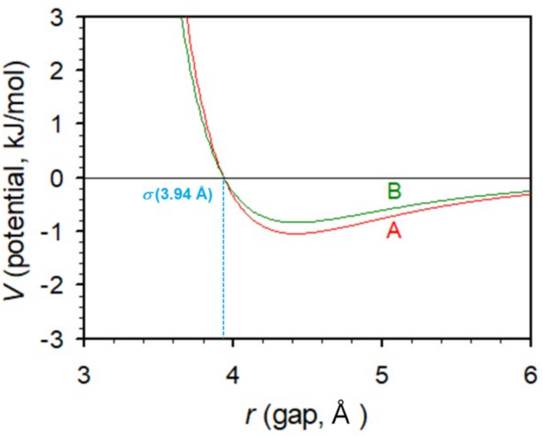

Fig. S6. Calculated Lennard-Jones potential parameters for *n*-alkanethiols ($CH_3(CH_2)_nSH$, *n*=even (A), and *n*=odd (B)). Consider the intermolecular potential (V, kJ/mol) between SAM and hexane probe separated by a distance of r (Å) using ε_{odd} (0.8315 kJ/mol), σ_{odd} (3.94 Å), ε_{even} (1.0393 kJ/mol) and σ_{even} (3.94 Å) according to the classical equation[8] of $V_{(r)} = 4 \times \varepsilon \left[\left(\frac{\sigma}{r}\right)^{12} - \left(\frac{\sigma}{r}\right)^{6} \right]$, where ε is the potential well depth, and σ is the distance where the potential equals zero (also double the van der Waals radius of the atom).

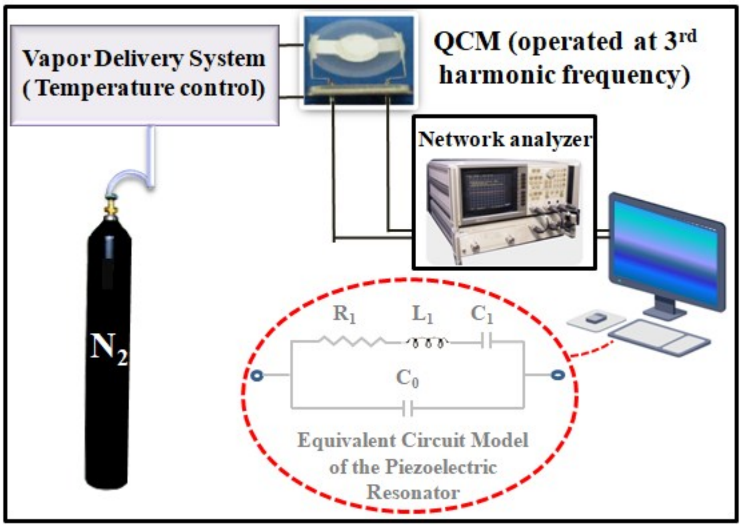

Scheme S3. The experimental setup of the QCM measurment using network analyzer for data acquisition and analysis.

References

[1] Z. Lin, M. D. Ward, *Anal. Chem*. 1996, **68**, 1285.
[2] S.W. Wenzel, R. M. White, *Appl. Phys. Lett*, 1989, **54**, 1976.
[3] O. Melroy, K. Kanazawa, J. G. Gordon, D. Buttry, *Langmuir*, 1986, **2**, 697.
[4] D. S. Karpovich, G. J. Blanchard. *Langmuir* 1994, **10**, 3315.
[5] M. Roshchina, N. K. Shonija, F. Bernardoni, A. Y. Fadeev. *Langmuir* 2014, **30**, 9355.
[6] K. P. Singh, D. Mohan, G. S. Tandon, G. S. D. Gupta. *Ind. Eng. Chem. Res*., 2002, **41**, 2480.
[7] L. Jiang, C. S. Sangeeth, C. A. Nijhuis, *J. Am. Chem. Soc*., 2015, **137**, 10659.
[8] H. Imahori, H. Norieda, Y. Nishimura, I. Yamazaki, K. Higuchi, N. Kato, T. Motohiro, H. Yamada, K. Tamaki, M. Arimura, Y. Sakata, *J. Phys. Chem. B*, 2000, **104**, 1253.
[9] Y. Feng, E. R. Dionne, V. Toader, G. Beaudoin, A. Badia, *J. Phys. Chem. C* 2017, **121**, 24626.
[10] B. Smit, S. Karaborni, J. I. Siepmann, *J. Chem. Phys.* 1995, **102**, 2126.
